# Unmet Needs of People with Severe Multiple Sclerosis and Their Carers: Qualitative Findings for a Home-Based Intervention

**DOI:** 10.1371/journal.pone.0109679

**Published:** 2014-10-06

**Authors:** Claudia Borreani, Elisabetta Bianchi, Erika Pietrolongo, Ilaria Rossi, Sabina Cilia, Miranda Giuntoli, Andrea Giordano, Paolo Confalonieri, Alessandra Lugaresi, Francesco Patti, Maria Grazia Grasso, Laura Lopes de Carvalho, Lucia Palmisano, Paola Zaratin, Mario Alberto Battaglia, Alessandra Solari, on behalf of the PeNSAMI project

**Affiliations:** 1 Unit of Psychology, Foundation IRCCS Istituto Nazionale per la Cura dei Tumori, Milan, Italy; 2 Department of Neuroscience, Imaging and Clinical Sciences, University “G. d'Annunzio” of Chieti-Pescara, Chieti, Italy; 3 Multiple Sclerosis Unit, Foundation IRCCS S. Lucia Rehabilitation Hospital, Rome, Italy; 4 Neurology Clinic, MS Center, University Hospital Policlinico Vittorio Emanuele, Catania, Italy; 5 AISM Liguria Region Rehabilitation Service, Genoa, Italy; 6 Unit of Neuroepidemiology, Foundation IRCCS Neurological Institute C. Besta, Milan, Italy; 7 Unit of Neuroimmunology, Foundation IRCCS Neurological Institute C. Besta, Milan, Italy; 8 Dept. of Therapeutic Research and Medicine Evaluation, Istituto Superiore di Sanità, Rome, Italy; 9 Scientific Research Department, Italian Multiple Sclerosis Foundation, Genoa, Italy; Brigham and Women's Hospital, Harvard Medical School, United States of America

## Abstract

**Background:**

Few data on services for people with severe multiple sclerosis (MS) are available. The Palliative Network for Severely Affected Adults with MS in Italy (PeNSAMI) developed a home palliative care program for MS patients and carers, preceded by a literature review and qualitative study (here reported).

**Objective:**

To identify unmet needs of people with severe MS living at home by qualitative research involving key stakeholders, and theorize broad areas of intervention to meet those needs.

**Method:**

Data were collected from: at least 10 personal interviews with adults with severe MS (primary/secondary progressive, EDSS≥8.0); three focus group meetings (FGs) of carers of people with severe MS; and two FGs of health professionals (HPs). Grounded theory guided the analysis of interview and FG transcripts, from which the areas of intervention were theorized.

**Results:**

Between October 2012 and May 2013, 22 MS patients, 30 carers and 18 HPs participated. Forty-eight needs themes were identified, grouped into 14 categories and four domains. Seven, highly interdependent intervention areas were theorized. Patients had difficulties expressing needs; experiences of burden and loneliness were prominent, chiefly in dysfunctional, less affluent families, and among parent carers. Needs differed across Italy with requirements for information and access to services highest in the South. All participants voiced a strong need for qualified personnel and care coordination in day-to-day home care. Personal hygiene emerged as crucial, as did the need for a supportive network and preservation of patient/carer roles within family and community.

**Conclusions:**

Unmet needs transcended medical issues and embraced organizational and psychosocial themes, as well as health policies. The high interdependence of the seven intervention areas theorized is in line with the multifaceted approach of palliative care. At variance with typical palliative contexts, coping with disability rather than end-of-life was a major concern of patients and carers.

## Introduction

In Western countries multiple sclerosis (MS) is second only to trauma as cause of chronic neurological disability in young adults. Around 15% of MS sufferers have a progressive course from the outset (primary progressive MS); a further 35% develop progressive disease after a variable period with relapsing-remitting course (secondary progressive MS) [1]. Although new therapies can attenuate disease course, people with primary or secondary progressive MS lack effective treatment options [2]. Reduced mobility is one of the commonest and most visible impairments of people with progressive MS, but concurrent compromise of other neurological functions, such as cognition, swallowing, and speech, are present in various combinations and impact each other [3]–[5]. Recent studies indicate that life expectancy of people with MS in general is reduced by about a decade [6]. However highly disabled patients may live many years [7], enduring co-morbidities and complications such as aspiration pneumonia, urinary tract infections, complications of falls and fractures, and sepsis secondary to pressure ulcers, all of which are major causes of death [8].

MS also impinges on the physical and psychological well-being of patients' significant others [9]. The long disease trajectory allows time for family members to prepare and adjust to their carer roles, which differ from those in cancer and other disabling neurological conditions such as stroke or amyotrophic lateral sclerosis. However, as time passes, carer ageing and co-morbidities add to the complexity and burden of the disease.

Although robust evidence supporting treatment decisions in advanced MS is lacking, recent guidelines suggest shifting to a palliative approach as the disease progresses [10]. Palliative care has been defined as: “The active total care of patients whose disease is not responsive to curative treatment. Management of pain and other symptoms, and of psychological, social and spiritual problems is paramount. The goal of palliative care is the achievement of the best quality of life for patients and their families.” (http://www.who.int/cancer/palliative/definition/en/accessed 1 April 2014).

Consequent European government initiatives, including Italian legislation of 2010 (http://www.normativasanitaria.it/jsp/dettaglio.jsp?id=32922 accessed 1 April 2014) aim to develop and improve palliative services for non-cancer patients including those with neurodegenerative diseases [11]–[14].

In this context, we have developed a new home-based intervention for people with severe MS and their carers called the Palliative Network for Severely Affected Adults with MS in Italy (PeNSAMI). We were inspired by two models: the phased approach for the development and evaluation of complex interventions proposed by the Medical Research Council [15]; and the approach envisaging interaction between neurology, rehabilitation and palliative care services as an effective way of managing patients with neurodegenerative disorders [16]. In developing PeNSAMI we specifically aimed to involve patients, caregivers and health professionals (HPs) in the development of the intervention; and take specific cultural, socioeconomic, and healthcare contexts across Italy into account [17].

From our systematic review of the literature (see below) we identified four publications [13], [14], [18], [19] and one unpublished study [20] which employed qualitative methodologies to assess MS patient needs, and were conducted in the UK, Germany and Italy over the last seven years. The UK [18], [19] and Italian [20] studies assisted the construction of a home palliative care service which was compared with standard care in phase II randomized controlled trials (RCTs). The UK RCT showed that a home palliative care service improved symptoms management, reduced caregiver burden and also reduced the use of primary and acute hospital services over the short-term [21]. The Italian RCT (Ne-Pal) included people with severe MS, Parkinson's disease and related disorders, or amyotrophic lateral sclerosis. They found that a home palliative care intervention produced a clinically and statistically significant improvement in patients' health-related quality of life and in four physical symptoms (pain, breathlessness, sleep, and bowel symptoms) [20].

Nevertheless it may not be straightforward to transfer interventions of this sort to different contexts and health systems [17], [22]. The UK RCT [21] was included in a recently published systematic review on the effectiveness and cost-effectiveness of home palliative care services for adults with advanced illness and their caregivers. The review found that home palliative care increased the chance of dying at home and reduced symptom burden especially for patients with cancer, without impacting on caregiver grief. However data on neurodegenerative conditions were few and heterogeneous [23].

Rehabilitation plays a major role in providing long-term MS care and support, often over many years, especially in the progressive phase of the disease: a systematic review on the effectiveness of multidisciplinary rehabilitation for adults with MS revealed improved activity and participation [24]. In caring for people with long-term neurological conditions such as MS, intensity of involvement and interaction between neurology, rehabilitation and palliative care depends on disease phase: as the patient's condition becomes more advanced, rehabilitation and palliative care approaches often overlap (neuropalliative rehabilitation) [16], and it is expected that exchange of competences and co-ordination between these specialities will improve care quality.

The present study reports the unmet needs perceived by severely affected MS adults, their carers, and HPs experienced in caring for them, as determined by the qualitative research method. The study is part of the PeNSAMI project which included a systematic review of the literature and the construction of a home-based palliative program to be tested in a multicenter setting spanning the three main geographic areas of Italy.

## Methods

### Data collection

We involved three key players: people with severe MS, their carers, and HPs experienced in the care of people with severe MS. Our objective was to explore their views, experiences and needs, and hence theorize the intervention areas.

For people with severe MS a home-based personal semi-structured interview methodology was felt to be most appropriate. For carers and HPs focus groups (FG) were held to promote interactions and exchange of ideas within a flexible structure [25]. Three FGs (one in each area of Italy) with carers of adults with severe MS, and two FGs with HPs (in Genoa and Rome) were conducted.

### Ethics statement

All study patients gave written or oral witnessed consent to participate in a personal audio-recorded interview. All carers and HPs gave written consent to participate in audio-recorded FGs. The protocol and consent procedures were approved by the Ethics Committee of: Foundation IRCCS Neurological Institute C. Besta, Milan; AISM Liguria Region Rehabilitation Service, Genoa; Foundation IRCCS S. Lucia Rehabilitation Hospital, Rome; University “G. d'Annunzio” of Chieti-Pescara, Chieti; and University Hospital Policlinico Vittorio Emanuele, Catania (all in Italy).

### Procedures

Five psychologists (all women, median age 35 [29–47], one at each participating center) conducted the personal semi-structured interviews in the homes of MS patients. Before the interview, data on patient's personal circumstances and general and clinical condition were available to interviewers via the study case report form compiled by the patient's caring neurologist. Patients had no relationship with the interviewer prior to the study. The psychologists were trained (one-day) to conduct the interview by two researchers (CB and EB) experienced in qualitative research in the fields of oncology and palliative care. A prototype interview guide was devised beforehand, based on the literature on severe MS [7], [8], [13], [14], [18]–[20], [22] and palliative care [26]. The guide was modified during the study based on the results emerging from the qualitative analysis. Each interview was audio-recorded and transcribed. Interview duration depended on the content of the patient's contribution and his/her willingness/ability to continue. Only exceptionally was a carer present (when the patient requested or appeared ill-at-ease in carer absence).

Each FG was planned to include 6–10 participants, plus 2 moderators. One moderator (EB, facilitator) had no previous contact with FG participants; her task was to engage participants, promote exchanges, modulate conflicts, and ensure that all the topics were adequately covered, while allowing time for exploration of any pertinent issues arising. The co-moderator (AS or EP) took notes, noted relevant non-verbal communication, assisted with logistics, and oversaw the audio recording. The FG guides were devised beforehand and modified as required. FG reports were sent to all participants for approval.

### Sampling and recruitment

Participants were selected using a purposeful sampling technique [27]. Those recruited for personal interviews were adults with definite MS [28], primary or secondary progressive form, Expanded Disability Status Scale [EDSS] [29] ≥8.0, who had a carer. Institutionalized patients, and those with severe cognitive compromise or impairment precluding communication were excluded. The intention was to recruit patients who varied in terms of age, gender, and intensity of care. A minimum of 10 interviews was planned; sampling ended when data saturation was achieved and no new themes emerged [25].

Participants in carer FGs were recruited from relatives, friends, next of kin, and “key decision makers” usually designated by the patient. Each FG had to include at least three carers of MS patients with severe cognitive compromise or inability to communicate. Participants in HP FGs were recruited from physicians, psychologists, nurses, social workers, and therapists, all involved in caring for patients with severe MS.

### Analysis

Grounded theory (constructivist approach) [30], guided the analysis of the interview and FG transcripts. We aimed to identify patients' unmet needs, and theorize broad areas of intervention to meet those needs. Two researchers (CB and EB) independently codified the raw transcripts. After joint discussion of interpretations, the researchers developed a coding framework. The constant comparative technique was used to identify emerging themes (open coding) [31]. Second-level (axial) coding was undertaken to group similar phenomena into categories [32]. Extensive notes were taken to trace researchers' thinking and guide conceptualization [33]. After the principal categories had been established, third level (selective) coding was undertaken [30], [34] to produce domains (at the highest level of abstraction).

The characteristics of patients to be recruited for subsequent interviews were decided based on the coding framework reported above; interviews were stopped when data saturation occurred (no new codes emerged).

## Results

### Participant characteristics

Between October 2012 and May 2013, 22 interviews were conducted at the five centers. Table 1 summarizes the characteristics of participants. Participant identification codes and background information are shown in Table S1. The personal interviews lasted on average 51 minutes (range 23 –102), and in two (9%) a paid carer also participated. No patient refused to participate.

**Table 1:** Characteristics of the 22 people with severe multiple sclerosis who participated in the personal semi-structured interviews.

Characteristic	Sub-characteristic	N (%)
Women		14 (64%)
Age (years)^1^		58.7, 9.3 (41–77)
Disease course	Primary progressive	6 (27%)
	Secondary progressive	16 (73%)
Time since MS diagnosis (years)^1^		22, 7.2 (14–39)
EDSS score^2^		9.0 (8.0–9.5)
PEG		2 (9%)
Barthel Index^2^		5 (0–55)
Marital status	Single	6 (27%)
	Married	13 (59%)
	Divorced	2 (9%)
	Widow	1 (5%)
Occupation	Employed	2 (9%)
	Home employment	1 (5%)
	Not working	19 (86%)
Living with	Family	9 (41%)
	Spouse	6 (27%)
	Mother (81, 85, 87 years)	3 (14%)
	Alone	2 (9%)
	Paid caregiver	2 (9%)
Followed by	MS center	11 (50%)
	Rehabilitation hospital	5 (23%)
	Rehabilitation service, community based	4 (18%)
	Neurologist	1 (5%)
	Family doctor	1 (5%)

MS is multiple sclerosis. EDSS is Expanded Disability Status Scale. PEG is percutaneous endoscopic gastrostomy.

1 Mean, SD (range).

2 Median (range).

The three carer FGs involved 30 participants (Table 2), lasted on average 126 minutes (range 120–140) and were conducted in Genoa, Rome and Catania. Carers were varied in terms of age and relation to the patient. Most were full-time caregivers, and six cared for MS patients with severe cognitive compromise. For many carers, participation in the FG was itself a challenge since a substitute carer was difficult to find, particularly for those from Northern and Central Italy; whereas finding a substitute (generally a relative) in the South was considerably easier. FGs were highly informative and emotionally rich, revealing many of the facets and challenges associated with the highly demanding caregiver role.

**Table 2:** Characteristics of the 30 carers who participated in the focus group meetings.

Characteristic	Sub-characteristic	N (%)
Women		16 (53%)
Age (years)^1^		59.2, 15.1 (24–91)
Relation to patient	Spouse	20 (66%)
	Parent	5 (17%)
	Offspring	3 (10%)
	Other relative	2 (7%)
Carer of patient with severe cognitive compromise		6 (20%)
Occupation	Home employment	7 (23%)
	Employed	10 (33%)
	Retired	13 (44%)
Caregiving for	>10 years	25 (83%)
	5–10 years	3 (10%)
	<5 years	2 (7%)
Time spent caregiving	Full-time	18 (60%)
	Part of the day	11 (37%)
	Part of the week	1 (3%)

1 Mean, SD (range).

The two HP FGs, one in Genoa and one in Rome, involved 18 participants (Table 3) and lasted 130 and 140 minutes. All the main professions involved in the care of people with severe MS were represented, although only one family doctor participated, in spite of efforts to find more. Nurses were the most common professionals (n = 4), followed by psychologists and physiotherapists (n = 3). Most participants had over 10 years of experience in MS. None come from the area of palliative care. All HPs contributed actively and were enthusiastic about the research aims.

**Table 3:** Characteristics of the 18 health professionals who participated in the focus group meetings.

Characteristic	Sub-characteristic	N (%)
Women		11 (62%)
Age (years)^1^		43.5, 10.2 (26–59)
Profession	Nurse	4 (22%)
	Physiotherapist	3 (16%)
	Psychologist	3 (16%)
	Physiatrist	2 (12%)
	Social worker	2 (12%)
	Neurologist	1 (5%)
	Occupational therapist	1 (5%)
	Speech therapist	1 (5%)
	Family doctor	1 (5%)
MS experience (years)	>10	11 (61%)
	5–10	4 (22%)
	<5	3 (17%)
No of MS patients followed in last 3 months	>30	6 (34%)
	10–30	9 (50%)
	<10	3 (16%)
No of severe MS patients followed in last 3 months	>20	1 (5%)
	10–20	9 (50%)
	<10	8 (45%)

MS is multiple sclerosis.

1 Mean, SD (range).

### Expressed needs

Forty-eight themes grouped into 14 categories and four domains emerged (Table 4).

**Table 4:** Codification of needs into themes, categories and domains, as referred by people with severe multiple sclerosis (**¥**), their carers (Δ) and health professionals (▴).

Domain	Category	Theme	Referred by
***“Managing everyday life”***	**Symptoms management**	Symptoms control	¥
		Physiotherapy	¥ Δ
		Devices/aids	¥
	**Personal care/hygiene**	Management	¥ Δ
		Reducing uneasiness, embarrassment	¥ Δ▴
		Professional for care	¥ Δ
	**Activities of daily living**	Reduce caregiver burden	¥ Δ▴
		More and better qualified professionals	¥ Δ▴
		Indoor mobility/housing adaptations/communication aids	¥ Δ▴
	**Outdoor mobility and transport**	Ability to get out	¥ Δ
		Equipment (elevators, stair-lifts, ramps, etc)	¥ Δ
		Aids that meet patient requirements	¥ Δ▴
***“Psychosocial”***	**Relationships/communication**	Preservation of family/social relationships	¥ Δ▴
		Not being alone at home	¥ Δ
		Relationships with other MS patients	¥ Δ
		Affection/empathy	¥ Δ▴
		Use of computer and social media	¥
		Reduce stigmatization	¥▴
	**Leisure/holidays**	Cultural activities	¥ Δ▴
		Getting out for leisure	¥ Δ▴
		Pets	¥
	**Psychological well-being/social role**	Dealing with fear of the (patient's) future	¥ Δ
		Being accepted	¥ Δ▴
		Preserve “normality”	¥ Δ▴
		Preserve respect	¥ Δ▴
		Preserve biographical continuity	¥ Δ▴
		Preserve family/social role and decisional autonomy	¥ Δ▴
		Being useful (to others)	¥
		Employment (of patients and caregivers)	¥ Δ▴
		Preserve hope with research and MS cure	¥Δ▴
		Psychological therapy	¥ Δ▴
***“Organization”***	**Information**	Entitlement to services and facilities	¥ Δ
		Competent persons/service	¥ ▴
	**Access to services**	Reduce bureaucracy	¥ Δ▴
		Help with affronting bureaucracy	¥ Δ▴
		Timely and sufficient delivery of aids/consumables	¥ Δ
		Services suited to people with severe MS	¥ Δ▴
	**Co-ordination of services**	Person to refer to (case manager)	Δ▴
		Networking	¥ Δ▴
	**Competent professionals**	Competent professionals	¥ Δ▴
***“Health and social policies”***	**Rights**	Criteria for benefit entitlement	¥ Δ
		Accessibility (reduction of architectural barriers)	¥ Δ▴
		Equity (across Italian areas)	¥ Δ
		Resource allocation, investment	¥ Δ▴
	**Culture**	Reduce stigmatization/discrimination	¥ Δ
		Respect for disabled rights/facilities	¥ Δ
	**Patient organizations**	Influence public health policies (lobbying)	Δ▴
		Promote initiatives (educational/cultural/leisure) and facilitate patient participation in them	¥ Δ

*Managing everyday life –* This domain comprised four categories, all of which were important for the daily life of the patient-caregiver dyad. Physiotherapy was an important theme: *Movement, I move little […], but a bit more physiotherapy during the week would be nice, yes that would be helpful* [Patient GIAD Genoa].

*Improving their quality of life would help them avoid degeneration… patients are sometimes abandoned as there is no cure for disease progression… so we must provide more and better physical therapy to maintain muscle tone* [Carer, FG Rome].

**Figure 1:**
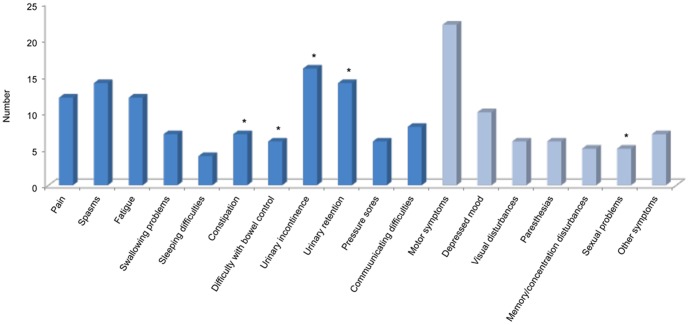
Histogram of symptoms (n = 145) reported by the 22 people with severe multiple sclerosis during the personal interviews. Blue bars identify symptoms of the Palliative Outcome Scale-Symptoms-Multiple Sclerosis [35]. The “other symptoms” category includes: shortness of breath (n = 2), peripheral edema (n = 2), thermoregulation problems (n = 2), and loss of smell (n = 1). Stars identify symptoms (n = 48, 33%) pertaining to the perineal area.

Most of the symptoms reported by patients revolved around bowel/bladder functions (Figure 1). Consequently, personal care and hygiene were of great concern to the dyad: *What we do most is cleaning up and things. These are what tire me and my caregiver the most. She has to turn me over… she says I've become heavier…* [Patient ALBU Genoa].

*I can't bring myself to ‘go’ in the diaper. I don't like being wet. I can't even go in the bedpan. I prefer the bathroom, but then they have to lift me. And I'm heavy, I know. But I try to help using my arms – more than that I can't manage*… [Patient MASA Milan].

*As regards my personal hygiene I am completely dependent… I have diapers and have to be changed… I can't do anything for myself. The diapers make me feel bad… I wish there was something else I could wear… like catheter or something invisible…* [Patient ASCO Chieti].

*I shower my daughter while she's in bed, there is no other way to do it*… [Carer, FG Genoa].

*I realize that I'm more trouble when I have a shower or a wash, or they have to change me. I always need help… That really kills me. I've stopped going out because I'm afraid of doing it in my pants… I'm always scared of sudden incontinence and creating a stink* [Patient GRRA Catania].

Managing these activities had strong implications, particularly for couples and family carers: *No, I can't wash myself, I've had to swallow my shame… I even had to get my son to do my bidet… That's hard…* [Patient TEDR Chieti].

*When I was working I would have to absent myself to help my wife go for a pee… I had to live with that… she didn't want anyone else to help her*… [Carer, FG Catania].

Home adaptations and mobility aids to enable leaving the house were a major concern; reasons for getting out were of secondary importance: *I need someone [to help]… at least then I would be a bit more independent… because leaving the house only 3 times a month isn't enough… I'm in jail… even prisoners are let out every day… Me no… and you see [how small] the house is…* [Patient ALSA Rome].

*What I really miss is getting out… no-one has time… It's better if I don't say how long it's been since I've been out… months. It's really really difficult* [Patient ASCO Chieti].

*My wife never leaves the house… she sleeps until the caregiver comes, mostly she watches television, eats and sleeps* [Carer, FG Genoa].

*Patients often ask us to help them get out, but they can't to tell us where they want to go… they just want to get out* [HP, FG Rome].

All players spoke of the need to alleviate the burden of the caregiver: *I need someone who can give my son [the caregiver] a break… It seems that I'm a burden, they tell me I'm not but* *…* [Patient GRRA Catania].

*It would help if caregiver had a bit of breathing space… time to get out, but [caregiver] doesn't have this and feels abandoned* [Carer, FG Rome].

*I think it's important for us to be able to get away for 5 minutes, although none of us readily admits it* [Carer, FG Catania].

*Psychosocial –* This was the largest domain, with 19 themes grouped into three categories: relationships/communication, leisure/holidays, and psychological wellbeing/social role. All players recognized the need to preserve relationships, and preserve the personal and social roles of the dyad (Table 4). Fear for the patient's future was a prominent theme, particularly for parent carers: *My mum is very old so… there is no future [for me]. I'm concerned that she's old, she's 85* [Patient ALBU Genoa].

*On Thursdays and Sundays it's my mother who has to look after me, even though she needs someone to help her… she's quite badly off… she's 87* [Patient MACI Rome].

*You worry about… [the patient]… thinking about when you're no longer here… I've made sacrifices to make sure my husband is OK if anything happens to me* *…* [Carer, FG Genoa].

*I'm fearful about when my husband and I are no longer here… we're both old… our relatives are all gone… what will happen to my son then?* [Carer, FG Genoa].

Social isolation and loneliness, as a consequence of disease worsening, had a negative effect on patient quality of life, particularly when carers were absent or dysfunctional: *I only want friendship, a chat. They don't have to stay for hours… 10 minutes would be enough… I just want someone to make me talk, make me laugh!* [Patient MASA Milan].

*Organization –* This domain included nine themes grouped into four categories: information, access to services, coordination of services, and competent professionals.

Organizational deficits and (obstructive) bureaucracy were experienced by all. Health and social services were described as scarce and difficult to access/obtain. The rehabilitation facilities that were available did not adequately meet the needs of people with severe MS. Patient aids and assistive devices emerged as a crucial issue with several facets: criteria and procedures for obtaining them; suitability for a given patient; difficulties patients have in accepting or familiarizing themselves with them. As was the case with health/social policies domain (see below), differences between different areas of Italy were enormous.

Obtaining information on patient rights and knowing what patients and their families were entitled to was another major problem for caregivers, whose impression was that practically no policies for the social protection for carers were in force or being implemented.

*By law we should have been reimbursed for the stair-lift we installed, but we never got anything… The money has always run out*… [Patient MAGH Chieti].

*We had to pay for the electric bed ourselves, even though they should have paid for it. I was cheated out of € 2100. I presented all the invoices but was never reimbursed, I don't know what to do about it* [Patient PASC Catania].

*We'd like to be informed about new regulations as they come out, and what we can apply for… we have to buy everything ourselves. They say that according to the law, we should get help, but it's not easy to get it* [Carer, FG Catania].

*I'd like more information about what [devices and aids] are available; we've only just found out about the existence of a baclofen pump*… [Carer, FG Genoa].

*What we are entitled to should be made public… available to everyone… not kept hidden*… [Carer, FG Genoa].

*You have to wait for ages on the phone, and then they can't give you an appointment for months or years… I used to get a check-up every six months, and have a blood test more easily. [Now there's] too much bureaucracy!* [Patient MAGH Chieti].

*The wheelchair is no longer comfortable… it's too tight. I have to keep asking them [my offspring] to make me more comfortable. At the clinic they say I'll have to wait 6 years for a new wheelchair* [Patient GRRA Catania].

*Health and social policies -* This domain consisted of nine themes grouped into three categories – rights, culture and patient organizations. All players referred to the need to reduce environmental barriers: *The sidewalks have been taken over by cars, you can't walk the sidewalk [you have to go round]. You go up, you go down, there are holes… you have to walk in road…* [Carer, FG Rome]

*We should set up strong [patients'] association, that looks after these questions on behalf of all of us with these problems* [Carer, FG Rome].

*We need a standard level of services in all districts; [services] shouldn't change depending on where you happen to live* [Carer, FG Rome].

The following citations refer to cultural issues: *We had to take [my condo] to court before we could install a stair-lift. They said it brought bad luck and looked bad. After we'd finally installed it, it got vandalized… What satisfaction when some residents asked us if they could use the stair-lift!* [Carer, FG Catania].

*When they come with a car they squeeze you in… you really feel you're disabled. If they come with a van, everyone knows [it's a disabled person]. It shouldn't be like that* [Patient ROPE Chieti]

### Death and sexuality/intimacy

*Death -* Patients never spontaneously broached death or end-of-life issues, which did not emerge as a primary need. The theme of death always had to be raised by interviewers, in accord with the interview guide.

*[Do you ever think about death?] Sometimes… [What do you think about?] That perhaps it will be a liberation* [Patient ALSA Rome].

Many patients expressed a wish not to outlive their carer (see psychosocial domain citations above, e.g. Patient ALBU Genoa).

Suicide and euthanasia were not mentioned by patients, who never presented openings for these issues to be explored by interviewers. It also emerged that patients had given little thought to advance care directives or end-of-life decisions.

*Sexuality and intimacy* - Some patients (mostly women) viewed loss of sexuality as a consequence of disease worsening: *You're invisible when you're disabled, you're not seen as normal… and [a sex life] is one of the many things you miss. But you've got to accept it. I suffered [when I lost my sexuality] just as I suffered when I lost my ability to walk. But I adapted, concentrating on other things. In that way I overcame it.*

[Patient FRLI Rome].

Some patients reported the need to adjust to functional limitations (including sexual functioning, incontinence, mobility compromise, and spasticity): *Some couples ask for help on how to manage intercourse, and these are couples that have maintained a form of intimacy* [HP, FG Genoa]. *Sometimes paid caregivers meet the sexual needs of male patients, or of the partners of women with MS* [HP, FG Rome].

Sexuality was strongly influenced by relational and patient-carer dynamics: *I haven't had sex for almost three years*. *I went to the doctor but what I need is a woman who wants to have sex… [My wife] has always been rather reluctant…* [Patient GIAD Genoa].

*My sex life* was so *short. When my MS became* serious *and I was no longer able to have intercourse, I cancelled men… denied everything… become completely asexual. Then thanks to a person I recovered some desire… and the desire has become quite strong… in short, now I've re-found myself* [Patient MACI Rome].

For partner carers, caregiving often extinguished intimacy as a result of the infantilization of the patient (see personal care and hygiene category above): *For young patients the disease stops them becoming adults, they remain at the stage of a child who is governed by the parents. But it can be like this even for adults*, *who once had independent and autonomous lives, but as the disease worsens, start being cared for by their elderly parents, and this brings the patient back to being a total child, who must silence [his/her]* *sexuality* [HP, FG Genoa].

The fact that the issue of sexuality never emerged during carer FGs may be due to the presence of other, more fundamental needs. However, addressing a more homogeneous population (e.g. partner carers) or using a different setting (e.g. individual interviews with carers) might have been more appropriate for allowing such an intimate theme to emerge.

In the HP FGs, sexuality emerged as difficult theme to approach with their patients: *Few patients speak about their sexual problems, so I do ask about them during consultations* [HP, FG Rome].

*Sex therapy is a taboo, it remains submerged. In critically ill patients it is not relevant, although it is in young patients without cognitive impairment* [HP, FG Rome].

### Carer and HP needs

During the FGs, important needs specific to carers and HPs emerged. Carers complained strongly about lack of time to themselves, and economic consequences for a family with a severely disabled person: *I can't get my wife out of the house. I get her into the lift, but there are six steps to negotiate so she changes her mind and doesn't want to leave. These are the problems. Sometimes I pay people to keep her company. Our offspring do not live close. I pay a lady to come. I pay a nurse to wash her in the morning. That's what I do. The [health services] don't send anyone, but by paying you get help. I worked to enjoy my retirement with my wife, but now that money is being spent [caring for her]. What else can I do?* [Carer, FG Genoa]

*Even those who have the money to pay, in the end they run out of savings because there are so many expenses* [Carer, FG Genoa].

Social relations and leisure time deprivation: *My old life has vanished. It's just me, my husband, and his disease. That's all. The disease stands between me and my husband. I used to work, now my life is dedicated to him. This is all very depressing and I don't know how long I can go on* [Carer, FG Genoa].

*I'm there day and night, but I think it's important to escape every now and then* [Carer, FG Catania].

Lack of time to attend medical appointments/procedures: *I need an operation on my shoulder, but who would take care of my husband? Then I would need to convalesce for two months… How am I going to solve this? I've phoned everywhere but no one will help because he's not self-sufficient* [Carer, FG Rome].

Carers were sometimes reluctant to leave their loved ones in the hands of someone else, who they felt may not be able to cope adequately. In addition, because attention was mainly focused on the patient, carers felt the need for their problems to be considered: they were all but forgotten and completely ignored by health policy makers.

*It's like having a baby, you go out for half an hour to do the shopping or something… but you always have this dread… you're never serene… you're always worried that something might happen while you're not there. Even if you take an hour off once a month, you never feel free* [Carer, FG Rome].

*You're afraid of getting sick, even of just a catching cold. I've experienced it: there are things that no one else can do, it doesn't matter how good the paid caregiver is, it's you who've known [the patient] since the beginning of the illness, only you know the little things you can do, that need to be done. You can't get sick. You can't even die, because then what would happen?* [Carer, FG Rome].

Many HPs reported difficulty in establishing a relationship with their MS patients: *Certain patients have an attitude that is conflictual or demanding… and this is a challenge for the professional* [HP, FG Genoa], or with the patient and his/her family: *When psychological distress disrupts the family, the operator must be even better at figuring out how to get around it… is very complicated* [HP, FG Genoa]. Sometimes carers tended to impose themselves between the patient and the HP, further inhibiting the patient: *[Sometimes] the caregiver needs more care than patient, because caregivers become another disease [burden] for their patients, sometimes they are harmful… they create a strong bond of dependency with the patient* [HP, FG Genoa].

Disproportionate expectations was another HP issue: *Patients expect us to work miracles or [give them] new treatments. It's difficult to make them see that we work for micro-objectives* [HP, FG Rome]. HPs strongly voiced the need for support in their demanding work with patients and their families: *We need psychological support for the difficult task we face, especially for home visits when family members [often] take advantage of our visit to let off steam* [HP, FG Genoa].

### Intervention areas

**Figure 2:**
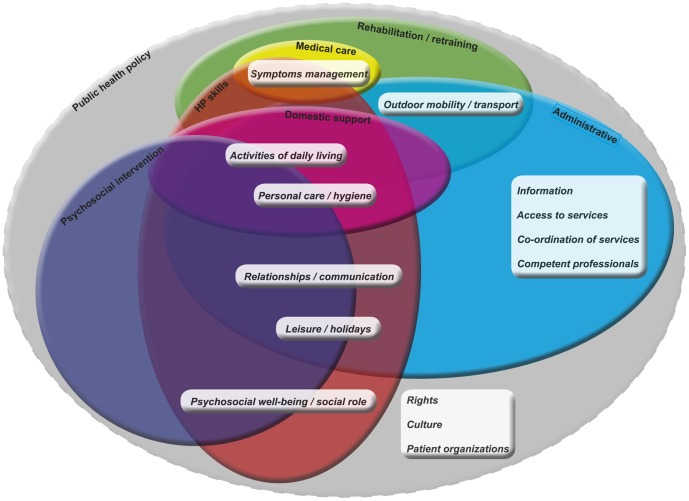
Venn diagram showing the seven intervention areas (medical care, yellow; rehabilitation/retraining, green; psychosocial interventions, blue; HP skills, orange; domestic assistance, purple; administration, light blue; public health policy, grey) labeled at the border of each ellipse. Each intervention area contains its related needs categories (in italics). The public health policy intervention area has a dashed border as it is not addressed in our palliative care program. HP is health professional.

In the last phase of the analysis, interventions to meet identified needs in the following seven areas were theorized: medical care (of physical symptoms due to MS and comorbidities); rehabilitation/retraining (physiotherapy and other interventions to improve/preserve patient functioning); psychosocial interventions (to improve psychological wellbeing and alleviate disease burden); HP skills (particularly communicational and relational); domestic support (for personal care/hygiene and other home-related issues); administrative (to improve accessibility to and coordination of services); and public health policies (pertaining to this population and addressing its specific needs). There was marked interdependence between these intervention areas, and the area of public health policies embraced all others (Figure 2).

## Discussion

The unmet needs reported by people with severe MS and their carers transcended purely medical issues to embrace psychosocial aspects. All participants voiced a strong need for qualified personnel and care coordination in the day-to-day home care and management of these patients. Personal care (particularly perineal hygiene) was viewed as crucial. Other priorities were mobility, the need for a supportive (social) network, and preservation of the patient's role within the family and the community. Coping with disability, rather than preparing for dying, emerged a major concern of patients, their carers, and HPs.

As also found by Galushko et al. [14] our patients, particularly the most disabled, had difficulty in articulating and deliberating on their needs, even though we purposely excluded those with severe cognitive compromise.

By contrast, carer FGs were rich in content and emotion, an indication that carers took seriously their responsibility as sole interpreter and provider of their loved ones' needs. However, these responsibilities had a profound impact on carer lives, particularly for parent carers and those in small or less affluent families. Feelings common to carers and patients were regret for the disruption of the original patient-carer relation, sense of isolation, and fear for the future.

A strength of our study is that a variety of stakeholders from the three geographic areas of Italy participated. Thus our results reflect a spectrum of perspectives and the social and cultural diversities that exist in Italy.

Availability of information about services, access to services, and appropriateness of services, varied with geography and were conspicuously worse in the South than the North. In general, families were larger in Central and Southern Italy so caregiving could be shared; in the North, by contrast, even participation in the carer FG often proved difficult. In the South however, cultural barriers and stigmatization could be as strong as physical and architectural obstacles (see for example the last two citations in the *health and social policies* paragraph of the Results).

Study limitations include the fact that issues of sexuality and intimacy were rarely raised by patients and never by their carers, although they were broached by HPs. This could be because carer FGs – whose participants were of variable age and relation to the patient – were unsuited for the discussion for such personal issues. Furthermore, although we invited family doctors to our HP FGs, only one attended. Similarly, although we wanted at least three carers of patients with severe cognitive compromise in each carer FG, we managed to recruit only two (however no additional themes emerged from these participants). Another limitation is that complementary and alternative medications were not part of the interview guide.

Many of the needs we identified were also reported by the qualitative studies of Edmonds et al. [18]; [19] and Galushko et al. [14], particularly the need to improve the continuity and coordination of care. Our study and the UK [18]; [19] study recruited MS patients of similar severity, while the German study [14] had few severely disabled patients. Our study differs from the others [14], [18], [19] in that it was multicentric, and did not originate from the palliative care field, but the MS field (Italian MS Society and MS clinicians and researchers, including those with expertise in MS rehabilitation). Notably, interviewers were trained by researchers who had oncology and palliative care backgrounds.

Nonetheless, the marked interdependence of the seven intervention areas we theorized is in line with the multifaceted approach of palliative care.

Our results were used to guide the construction of our home-based palliative program, which addresses each of the intervention areas identified in our analysis except the all-important area of public health policy. Our next step will be to assess the effectiveness of the home-based palliative program in a randomized controlled trial (ISRCTN73082124) and a qualitative study nested in the trial.

## Supporting Information

Table S1 **Background characteristics of the 22 interviewed people with multiple sclerosis.**
(DOC)
