# Supplementary material for: Unmet Needs of People with Severe Multiple Sclerosis and Their Carers: Qualitative Findings for a Home-Based Intervention
Source: PLoS One. 2014 Oct 6;9(10):e109679. doi: 10.1371/journal.pone.0109679 (PMC4186842; doi:10.1371/journal.pone.0109679)
Supplement: Table S1 — Background characteristics of the 22 interviewed people with multiple sclerosis. (DOC) [file pone.0109679.s001.doc]

**Table S1.** Background characteristics of the 22 interviewed people with multiple sclerosis.

| Identification  code | Center | Sex | Age (years) | Marital status | Living with | Occupation | EDSS |
| --- | --- | --- | --- | --- | --- | --- | --- |
| ALBU | Genoa | Man | 54 | Divorced | Mother (85 y) | Home employment | 9.0 |
| ALLI | Catania | Man | 41 | Single | Parents, a brother | Disability pension | 8.5 |
| ALSA | Rome | Man | 55 | Married | Wife | Disability pension | 9.0 |
| ASCO | Chieti | Woman | 62 | Married | Husband | Disability pension | 8.5 |
| BRRO | Rome | Man | 56 | Divorced | Paid caregiver | Clerk, full time | 9.5 |
| CLNA | Milan | Woman | 59 | Single | Paid caregiver | Disability pension | 9.5 |
| FRLI | Rome | Woman | 54 | Married | Husband, 2 adult children, paid caregiver | Disability pension | 9.0 |
| GIAD | Genoa | Man | 65 | Married | Wife | Disability pension | 8.5 |
| GIVI | Catania | Woman | 65 | Married | Husband | Housewife | 8.5 |
| GRRA | Catania | Woman | 61 | Widow | Alone | Disability pension | 8.5 |
| GUBA | Milan | Man | 60 | Married | Wife, a daughter | Disability pension | 8.5 |
| LIRU | Chieti | Woman | 77 | Married | Husband | Retired | 8.0 |
| MACI | Rome | Woman | 64 | Single | Mother (87 y) | Disability pension | 9.0 |
| MAGH | Chieti | Woman | 55 | Married | Husband, 2 adult children | Disability pension | 9.0 |
| MASA | Milan | Woman | 76 | Married | Husband, paid caregiver | Disability pension | 8.0 |
| PASC | Catania | Man | 41 | Single | Parents, a brother | Disability pension | 8.0 |
| PATU | Milan | Woman | 53 | Single | Mother (81 y) | Clerk, part-time | 8.0 |
| ROPE | Chieti | Man | 50 | Married | Wife, 3 adult children | Disability pension | 9.0 |
| SABE | Genoa | Woman | 56 | Married | Husband | Housewife | 9.0 |
| SIMO | Genoa | Woman | 72 | Single | Alone | Disability pension | 9.0 |
| TECA | Rome | Woman | 52 | Married | Husband, 2 adult children | Disability pension | 9.5 |
| TEDR | Chieti | Woman | 64 | Married | Husband, daughter and her partner | Disability pension | 9.0 |

EDSS is Expanded Disability Status Scale.
